# Family Socioeconomic Status and Neurodevelopment Among Patients With Dextro-Transposition of the Great Arteries

**DOI:** 10.1001/jamanetworkopen.2024.45863

**Published:** 2024-11-19

**Authors:** Adam R. Cassidy, Valerie Rofeberg, Emily M. Bucholz, David C. Bellinger, David Wypij, Jane W. Newburger

**Affiliations:** 1 Department of Psychiatry and Psychology, Mayo Clinic, Rochester, Minnesota; 2 Department of Pediatric and Adolescent Medicine, Mayo Clinic, Rochester, Minnesota; 3 Department of Cardiology, Boston Children’s Hospital, Boston, Massachusetts; 4 Section of Cardiology, Department of Pediatrics, University of Colorado School of Medicine, Aurora; 5 Department of Neurology, Boston Children’s Hospital, Harvard Medical School, Boston, Massachusetts; 6 Department of Psychiatry and Behavioral Sciences, Boston Children’s Hospital, Harvard Medical School, Boston, Massachusetts; 7 Department of Pediatrics, Harvard Medical School, Boston, Massachusetts; 8 Department of Biostatistics, Harvard T.H. Chan School of Public Health, Boston, Massachusetts

## Abstract

**Importance:**

Data are limited on the longitudinal implications of socioeconomic status (SES) for neurodevelopmental outcomes among persons with complex congenital heart disease (CHD).

**Objectives:**

To examine the association of family SES, maternal educational level, and maternal IQ with the neurodevelopment of individuals with dextro-transposition of the great arteries (d-TGA) from age 1 to 16 years and to identify how SES-related disparities change with age.

**Design, Setting, and Participants:**

This cohort study analyzed data of participants enrolled in the Boston Circulatory Arrest Study, a randomized clinical trial conducted in Boston, Massachusetts, from 1988 to 1992. Participants were infants with d-TGA who underwent arterial switch operation and, after operation, underwent in-person neurodevelopmental status evaluations at ages 1, 4, 8, and 16 years. Analyses were conducted from April 2021 to August 2024.

**Exposures:**

Mean Hollingshead scores at birth, age 1 year, and age 4 years were used to assign participants to SES tertiles (lowest, middle, or highest).

**Main Outcomes and Measures:**

Age-appropriate neurodevelopmental outcomes assessed at 4 study time points (ages 1, 4, 8, and 16 years) via in-person administration of a range of well-validated measures. Standardized neurodevelopmental composite scores from each evaluation were derived from principal component analysis and compared across SES tertiles, adjusting for birth and medical characteristics. These scores were used to categorize the sample into latent classes; patient and medical factors for a 3-class model were used to estimate latent class using multinomial regression.

**Results:**

The sample included 164 patients with d-TGA (123 males [75%]; mean [SD] gestational age at birth, 39.8 [1.2] weeks; 3 with Asian [2%], 6 with Black [4%], 5 with Hispanic [3%], and 146 with White [89%] race and ethnicity) and their mothers (mean [SD] age at birth, 28.5 [5.2] years). Lower SES tertile was associated with worse scores on most individual neurodevelopmental tests and worse neurodevelopmental composite scores at ages 4, 8, and 16 years. For example, mean (SD) neurodevelopmental composite scores at age 4 years were –0.49 [0.83] for lowest, 0.00 [0.81] for middle, and 0.47 [1.10] for highest SES tertile (*F*_2_ = 15.5; *P* < .001). When measured at consecutive time points, differences between SES tertiles were of similar magnitude. A latent class analysis produced 2- and 3-class models representing patients with stable (103 [64%] and 85 [53%]), improving (20 [13%]), and declining (57 [36%] and 55 [34%]) neurodevelopmental status. Those experiencing declines in neurodevelopmental status were more likely to have younger maternal age at childbirth (26.6 [5.1] vs 29.6 [4.9] and 29.1 [5.1] years; *P* = .002), lower maternal IQ (91.0 [14.1] vs 100.1 [11.1] and 96.2 [11.0]; *P* < .001), and lower SES (35.2 [10.8] vs 40.9 [9.9] and 35.8 [10.1]; *P* = .003) compared with those with stable or improving status.

**Conclusions and Relevance:**

This cohort study of individuals with d-TGA found an association between lower family SES and worse neurodevelopmental outcomes in childhood and continuing throughout adolescence as well as greater decline in neurodevelopmental status over time. Effective strategies are needed to improve access to neurodevelopmental monitoring and intervention services for children with CHD from lower socioeconomic backgrounds.

## Key Points

**Question:**

How is socioeconomic status (SES) associated with the neurodevelopment over time of individuals with dextro-transposition of the great arteries (d-TGA)?

**Findings:**

In this cohort study of 164 patients with d-TGA evaluated at 1, 4, 8, and 16 years, lower SES was associated with worse neurodevelopmental outcomes from early childhood through adolescence. Declining neurodevelopmental status over time was associated with lower SES tertile and younger maternal age at childbirth.

**Meaning:**

Findings of this study suggest that neurodevelopmental monitoring and intervention services should be intensified for children with critical congenital heart disease, such as d-TGA, and low SES.

## Teaser

This cohort study evaluates the association between socioeconomic status and neurodevelopment throughout childhood and adolescence among individuals with dextro-transposition of the great arteries (d-TGA), a complex congenital heart disease.

## Introduction

Decades of research attest to the neurodevelopmental, neuropsychological, and psychosocial (hereafter, referred to collectively as neurodevelopmental) risks associated with congenital heart disease (CHD) survivorship.^1,2,3^ While some children born with even the most complex forms of CHD have no discernable neurodevelopmental sequelae, other children endure substantial morbidities from neurological injury, cognitive deficits, psychosocial stressors, and/or diminished independence.^4^ Research on factors associated with neurodevelopmental outcomes in individuals with CHD has evolved from focusing on medical-surgical risk factors to incorporating social determinants of health.

Family socioeconomic status (SES) consistently ranks among the strongest correlates of health and well-being in the general population^5^ and those with chronic medical conditions. Children with CHD who are from socioeconomically disadvantaged backgrounds are less likely to be diagnosed in utero,^6,7,8,9^ are at higher risk for early-life^10^ and posttransplant mortality,^11^ experience more surgical complications^7^ and longer lengths of stay in the hospital,^12,13^ require more frequent hospital readmissions,^14^ and report lower health-related quality of life^15^ than their counterparts with higher SES. Additionally, children and adolescents with CHD are at greater risk for neurodevelopmental impairment across a range of domains, including general cognitive ability, language or communication, executive function, and memory.^16,17,18,19,20,21,22,23,24,25^ Current understanding of how SES affects neurodevelopmental outcomes over time has been hampered by a paucity of studies with high-quality longitudinal SES and outcomes data.

In this study, we aimed to examine the association of family SES, maternal educational level, and maternal IQ with the neurodevelopment of individuals with dextro-transposition of the great arteries (d-TGA) from age 1 to 16 years and to identify how SES-related disparities change with age. We hypothesized that SES-related disparities in neurodevelopmental outcomes would become apparent by 4 years of age, that SES-related disparities in neurodevelopmental outcomes would become more pronounced across childhood and adolescence, and that higher SES would mitigate the adverse implications of known medical and surgical risk factors for neurodevelopmental outcomes.

## Methods

### Study Design

We analyzed data of participants in the Boston Circulatory Arrest Study (BCAS), as previously described.^18,19,26,27,28,29^ Briefly, the BCAS was a randomized clinical trial, which was conducted from 1988 to 1992, to examine the incidence of brain injury among infants with d-TGA after assignment to either deep hypothermia with low-flow cardiopulmonary bypass or total circulatory arrest as their predominant method of vital organ support during the arterial switch operation (ASO).^26^ The Boston Children's Hospital Institutional Review Board approved all phases of the BCAS. Written informed consent was provided by parents or guardians at each phase, and participants aged 16 years provided assent. The present cohort study was part of the BCAS, which included an aim to look at longitudinal trajectory, for which informed consent was obtained. We followed the Strengthening the Reporting of Observational Studies in Epidemiology (STROBE) reporting guideline.

Eligible participants included infants with d-TGA (with or without ventricular septal defect) who were scheduled for surgical repair with the ASO before 3 months of age. Infants who weighed less than 2.5 kg at birth, had a suspected genetic syndrome or other extracardiac anomalies, had previous cardiac surgery, or had anomalies requiring aortic arch reconstruction or additional open heart operations were excluded. There was no selection bias by race and ethnicity among patients whose parent or guardian consented to enrollment in the BCAS (94% of eligible patients were enrolled). After hospital discharge, neurodevelopmental status was evaluated at 4 time points: ages 1, 4, 8, and 16 years. Maternal data and cognitive data were collected during the course of the BCAS. Participants who resided outside the US were not invited to return after age 1 year. Among 171 eligible participants who underwent the ASO, 155 were evaluated at age 1 year, 158 at age 4 years, 155 at age 8 years, and 139 at age 16 years (eFigure 1 in Supplement 1).

The primary end point was age-appropriate neurodevelopmental status. This status was assessed at each study time point using in-person administration of well-validated measures.^30,31,32,33,34,35,36,37,38^ eTable 1 in Supplement 1 provides a list of the measures used and an explanation of what the highest scores for each measure indicate.

### Variable Definitions

Demographic and medical characteristics were ascertained as previously described.^18,19,26,27,28,29^ Family SES was measured at each study time point using the Hollingshead Four Factor Index of Social Status (score range: 8-66, with the highest score indicating higher family SES; AA Hollingshead, unpublished manuscript, 1975). To obtain a global picture of family SES that spanned the critical early childhood years, we calculated an unweighted mean of Hollingshead scores, collected at birth, age 1 year, and age 4 years, to assign participants to SES tertiles (lowest, middle, or highest). Maternal IQ was estimated using the Peabody Picture Vocabulary Test–Revised^39^ (score range, 20-160; highest score indicates better single-word receptive vocabulary).

### Statistical Analysis

Birth, medical, and sociodemographic characteristics were compared across SES tertiles, maternal educational levels, and maternal IQ tertiles using Cochrane-Armitage trend tests for binary demographic variables and Jonckheere-Terpstra trend tests for all other variables. Analysis of covariance was used to compare neurodevelopmental outcomes by SES tertiles, maternal educational levels, and maternal IQ tertiles after adjusting for selected birth and medical characteristics: gestational age, birth weight, White vs other (Asian, Black or African American, Hispanic or Latinx, and other [multiracial and unknown for the lowest SES tertile, and multiracial for the highest SES tertile]) race and ethnicity (due to small counts), ventricular septal defect, total deep hypothermic circulatory arrest time and total support time, time from first surgery to discharge, and clinical seizure. Race and ethnicity data were self-reported by participants; these data were collected as a requirement of National Institutes of Health funding and as a means of gauging potential selection bias in BCAS participants vs nonparticipants at each stage of the study.

Because quantitative trends were similar for SES, maternal educational level, and maternal IQ, we focused subsequent analyses on SES. To account for repeated measures, generalized estimating equations with the exchangeable working correlation assumption were used when comparing outcomes across time points. To account for multiple pairwise comparisons, a Bonferroni-corrected 2-sided *P* < .0167 was considered statistically significant when comparing 3 groups.

Principal component analysis (PCA) was performed at each study time point using the regularized iterative PCA algorithm to accommodate missing values based on selected neurodevelopmental measures. The first principal component was then used to derive a standardized neurodevelopmental composite score with a mean (SD) of 0 (1), representing overall neurodevelopmental status at each time point. Analysis of covariance was used to compare neurodevelopmental composite scores by SES tertiles after adjusting for selected birth and medical characteristics.

The neurodevelopmental composite scores were then used to categorize the sample into latent classes, assuming an ellipsoidal mixture with equal volume, shape, and orientation. The probability of being assigned to a given latent class was estimated for all participants with 2 or more study evaluations, and participants were assigned to the latent class with the highest probability.^40^ Birth, medical, and sociodemographic characteristics were then used to estimate latent class using logistic or multinomial regression. Analyses were conducted from April 2021 to August 2024 using the base, missMDA, and mclust packages in R, version 3.6.0 (R Project for Statistical Computing).

## Results

The sample included 164 patients with d-TGA (123 males [75%], 41 females [25%]; mean [SD] gestational age at birth, 39.8 [1.2] weeks; 3 with Asian [2%], 6 with Black [4%], 5 with Hispanic [3%], 146 with White [89%], and 4 with other [2%] race and ethnicity), of whom 54 were in the lowest, 55 in the middle, and 55 in the highest SES tertiles; their mothers had a mean [SD] age at birth of 28.5 (5.2) years. Table 1 presents participant birth, medical, and sociodemographic characteristics by SES tertile. Participants in the lowest SES tertile (median [range] Hollingshead score, 28.0 [12.0-33.3]) had lower birth weights and were more likely to identify as races other than White than participants in the middle (median [range] Hollingshead score, 39.0 [33.4-43.3]) or highest (median [range] Hollingshead score, 49.7 [43.4-57.0]) SES tertiles. Maternal age, educational level, and IQ were higher among participants in the highest vs other 2 SES tertiles. Patients in each SES tertile were comparable in gestational age, birth weight, total deep hypothermic circulatory arrest time and total support time during the ASO, ventricular septal defect percentage, clinically apparent seizure history, and reoperation rate. Participants who did not return for evaluation at ages 1, 4, 8, and 16 years did not differ from those who returned in terms of continuous SES score, SES tertile, maternal IQ, maternal age, or maternal educational level at any age.

**Table 1.** Birth, Medical, and Sociodemographic Characteristics by SES Tertile

Characteristic	SES tertile, No. (%)			*P* value^a^
	Lowest (n = 54)	Middle (n = 55)	Highest (n = 55)	
Birth				
Gestational age at birth, mean (SD), wk	39.6 (1.4)	39.8 (1.0)	39.9 (1.2)	.38
Birth weight, mean (SD), kg	3.4 (0.4)	3.6 (0.5)	3.6 (0.4)	.04
Sex				
Female	16 (30)	11 (20)	14 (25)	.62
Male	38 (70)	44 (80)	41 (75)	
Race and ethnicity^b^				
Asian	0	2 (4)	1 (2)	.01
Black or African American	5 (9)	0	1 (2)	
Hispanic or Latinx	4 (7)	1 (2)	0	
White	43 (80)	52 (95)	51 (93)	
Other^c^	2 (4)	0	2 (4)	
Medical				
Ventricular septal defect	13 (24)	11 (20)	14 (25)	.86
Total DHCA time, mean (SD), min	36 (23)	35 (21)	37 (22)	.67
Total support time, mean (SD), min	145 (39)	141 (20)	143 (31)	.84
Time from first surgery to discharge, mean (SD), d	12 (12)	10 (4)	10 (5)	.52
Clinical seizure	2 (4)	5 (9)	4 (7)	.46
Reoperation by age 16 y	5 (9)	7 (13)	4 (7)	.72
Sociodemographic				
Maternal age at birth, mean (SD), y	26.0 (5.5)	28.3 (4.8)	31.2 (3.7)	<.001
Maternal educational level				
≤High school diploma	32 (59)	11 (20)	2 (4)	<.001
Some college	15 (28)	19 (35)	5 (9)	
College degree or graduate school	7 (13)	25 (45)	48 (89)	
Maternal IQ, mean (SD)	88 (14)	98 (10)	103 (10)	<.001

Abbreviations: DHCA, deep hypothermic circulatory arrest; SES, socioeconomic status.

^a^ *P* values comparing SES tertiles were calculated by Cochrane-Armitage trend test for binary variables, Fisher exact test for race and ethnicity, and Jonckheere-Terpstra trend test for all other variables.

^b^ Race and ethnicity data were self-reported by participants in the Boston Circulatory Arrest Study.

^c^ Other race and ethnicity included multiracial and unknown for the lowest SES tertile and multiracial for the highest SES tertile.

### Neurodevelopmental Status

Table 2 and eTable 2 in Supplement 1 show neurodevelopmental outcomes stratified by SES tertile at each time point and adjusted for selected birth and medical characteristics. Scores on the Bayley Scales of Infant and Toddler Development at age 1 year did not differ across SES tertiles. Mean (SD) neurodevelopmental composite scores at age 1 year were lower in the lowest SES tertile compared with the combined middle and high SES tertiles after adjustment for selected birth and medical characteristics (–0.26 [1.11] vs 0.14 [0.91]; *P* = .01). The association of neurodevelopmental performance with SES was of greater magnitude at later ages. Specifically, at the 4-, 8-, and 16-year time points, significant differences in scores were observed across SES tertiles on nearly all measures, including the neurodevelopmental composite scores (eg, mean [SD] scores at age 4 years: –0.49 [0.83] for lowest, 0.00 [0.81] for middle, and 0.47 [1.10] for highest SES tertile; *F*_2_ = 15.5; *P* < .001). No differences in scores were found with the following measures: the Grooved Pegboard Test at age 4 years, the Trail Making Test (trail B minus trail A) at age 8 years, and the Numbers subtest of the Children’s Memory Scale and the General Executive Composite of the Behavior Rating Inventory of Executive Function at age 16 years.

**Table 2.** Neurodevelopmental Outcomes at Ages 1, 4, 8, and 16 Years by SES Tertile

Neurodevelopmental measure^a^	Scores by SES tertile, mean (SD)			Adjusted *P* value^b^
	Lowest (n = 54)	Middle (n = 55)	Highest (n = 55)	
Age 1 y				
PDI^c^	91.5 (17.4)	97.5 (15.1)	95.6 (14.5)	.09
MDI^c^	101.0 (16.6)	106.4 (14.2)	107.0 (15.4)	.12
Neurodevelopmental composite score at 1 y	–0.26 (1.11)	0.16 (0.91)^d^	0.12 (0.92)^d^	.04
Age 4 y				
FSIQ^c^	85.1 (11.1)	92.1 (12.3)^e^	100.3 (16.3)^e^^,^^f^	<.001
Verbal IQ	88.6 (10.2)	93.7 (14.8)	102.7 (16.0)^e^^,^^f^	<.001
Performance IQ	84.5 (12.4)	92.2 (11.6)^e^	97.7 (15.8)^e^	<.001
ROWPVT	88.0 (15.4)	99.1 (14.9)^e^	103.1 (12.8)^e^	<.001
EOWPVT^c^	84.9 (11.3)	91.9 (13.7)	99.8 (17.5)^e^^,^^f^	<.001
Grooved Pegboard Test^c^	99.9 (43.5)	88.1 (34.0)	87.7 (39.9)	.24
Neurodevelopmental composite score at 4 y	–0.49 (0.83)	0.00 (0.81)^e^	0.47 (1.10)^e^^,^^f^	<.001
Age 8 y				
FSIQ^c^	89.0 (12.6)	97.4 (12.3)^e^	104.6 (16.4)^e^^,^^f^	<.001
Verbal IQ	91.9 (13.4)	99.8 (15.1)^e^	107.1 (17.5)^e^	<.001
Performance IQ	88.0 (13.4)	95.3 (11.3)^e^	101.2 (14.9)^e^	<.001
TOVA Errors of Commission^c^	14.7 (19.2)	12.3 (15.1)	7.7 (11.0)^e^	.06
DGS^c^	8.2 (2.3)	8.7 (2.6)	9.5 (2.8)^e^	.02
TMT B minus A^c^	64.7 (58.6)	60.1 (57.6)	46.1 (40.0)	.12
WRAML Memory Screening Index^c^	84.4 (13.2)	86.6 (12.9)	98.8 (15.9)^e^^,^^f^	<.001
Grooved Pegboard Test^c^	106.0 (38.6)	100.2 (26.0)	92.8 (19.8)^e^	.01
WIAT Reading^c^	88.3 (12.2)	95.7 (15.2)^e^	101.6 (14.8)^e^	<.001
WIAT Mathematics^c^	88.2 (14.4)	98.6 (14.9)^e^	102.6 (18.8)^e^	<.001
Neurodevelopmental composite score at 8 y	–0.49 (0.79)	–0.03 (0.91)^e^	0.49 (1.04)^e^^,^^f^	<.001
Age 16 y				
D-KEFS Average	8.2 (1.8)	9.0 (2.1)^e^	9.7 (2.1)^e^	<.001
D-KEFS Inhibition^c^	7.5 (3.0)	7.5 (3.7)	9.2 (3.6)^e^	.03
D-KEFS Number-Letter Switching^c^	7.8 (3.3)	7.7 (3.5)	9.3 (3.0)	.03
CMS General Memory^c^	84.5 (18.5)	88.9 (17.3)	97.1 (17.8)^e^	.004
CMS Numbers Total Score^c^	7.4 (2.8)	7.7 (3.7)	8.5 (3.3)	.20
BRIEF-Parent Global	55.4 (12.0)	58.0 (12.1)	51.4 (11.7)^e^	.03
WIAT-II Reading^c^	88.2 (13.3)	94.4 (15.0)^e^	103.1 (16.0)^e^^,^^f^	<.001
WIAT-II Mathematics^c^	86.8 (18.0)	98.8 (18.2)^e^	103.3 (19.5)^e^	<.001
Neurodevelopmental composite score at 16 y	–0.38 (0.82)	–0.09 (0.95)	0.41 (1.06)^e^	<.001

Abbreviations: BRIEF, Behavior Rating Inventory of Executive Function (highest score indicating more parent-reported executive function problems); CMS, Children’s Memory Scale (highest score indicating better learning and memory abilities); DGS, Digit Span (highest score indicating better auditory attention and working memory); D-KEFS, Delis-Kaplan Executive Function System (highest score indicating better executive function skills); EOWPVT, Expressive One-Word Picture Vocabulary Test (highest score indicating better expressive vocabulary); FSIQ, Full Scale IQ; MDI, Mental Development Index (highest score indicating better early memory, language, and problem-solving abilities); PDI, Psychomotor Development Index (highest score indicating better gross and fine motor skills); ROWPVT, Receptive One-Word Picture Vocabulary Test (highest score indicating better receptive vocabulary); SES, socioeconomic status; TMT, Trail Making Test (highest score indicating worse cognitive flexibility); TOVA, Test of Variables of Attention (highest score indicating higher frequency of impulsive errors); WIAT, Wechsler Individual Achievement Test (highest score indicating better academic skills); WRAML, Wide Range Assessment of Memory and Learning (highest score indicating better learning and memory abilities).

^a^ eTable 1 in Supplement 1 provides the score ranges for all measures used.

^b^ *P* values with 2 *df* compared neurodevelopmental composite scores by SES tertiles and were calculated by analysis of covariance adjusted for selected birth and medical characteristics.

^c^ Contribution to the neurodevelopmental composite score.

^d^ *P* = .01 for comparison of neurodevelopmental composite scores by combined middle and highest SES tertiles vs lowest SES tertile.

^e^ *P* < .0167 for comparison with the lowest SES tertile.

^f^ *P* < .0167 for comparison with the middle SES tertile.

At the 4-, 8-, and 16-year time points, neurodevelopmental composite scores were approximately 0.5 SD better for the middle SES tertile compared with the lowest SES tertile (eg, age 8 years: –0.03 [0.91] vs –0.49 [0.79]) and nearly 1.0 SD better for the highest SES tertile compared with the lowest SES tertile (eg, age 8 years: 0.49 [1.04] vs –0.49 [0.79]) (Table 2 and eTable 2 in Supplement 1). At these time points, similar-sized differences were found between the highest and lowest maternal IQ tertiles (eg, age 8 years: 0.40 [1.03] vs –0.43 [1.00]) as well as between the highest and lowest maternal educational levels (eg, age 8 years: 0.30 [1.04] vs –0.33 [0.89]) (eTable 3, eTable 4, and eFigure 2 in Supplement 1).

Pairwise comparisons of the SES tertiles revealed 3 distinct patterns. In the first pattern, significant differences in trend were noted between each SES tertile (eg, mean [SD] Full Scale IQ [FSIQ] at age 4 years: 85.1 [11.1] for lowest vs 92.1 [12.3] for middle vs 100.3 [16.3] for highest). Other neurodevelopmental outcomes with this pattern included the FSIQ at age 8 years, Expressive One-Word Picture Vocabulary Test at age 4 years, and Verbal and Performance IQ at 8 years of age and Reading at age 16 years. In the second pattern, the middle and lowest SES tertiles did not differ from one another, but both were significantly lower than the highest SES tertile (eg, mean [SD] Verbal IQ at age 4 years: 88.6 [10.2] for lowest vs 93.7 [14.8] for middle vs 102.7 [16.0] for highest). Other neurodevelopmental outcomes with this pattern included Expressive One-Word Picture Vocabulary Test at age 4 years, FSIQ, Verbal IQ, and Performance IQ at age 8 years, and Reading at age 16 years. In the third pattern, the middle and highest SES tertiles did not differ from one another, but both were significantly higher than the lowest SES tertile (eg, mean [SD] Receptive One-Word Picture Vocabulary Test score at age 4 years: 88.0 [15.4] for lowest vs 99.1 [14.9] for middle vs 103.1 [12.8] for highest). Other neurodevelopmental outcomes with this pattern included the Wechsler Individual Achievement Test (WIAT) Reading at age 8 years, WIAT Mathematics at ages 8 and 16 years, and Delis-Kaplan Executive Function System Average at age 16 years.

### Change in SES-Related Neurodevelopmental Disparities Over Time

**Figure 1.**
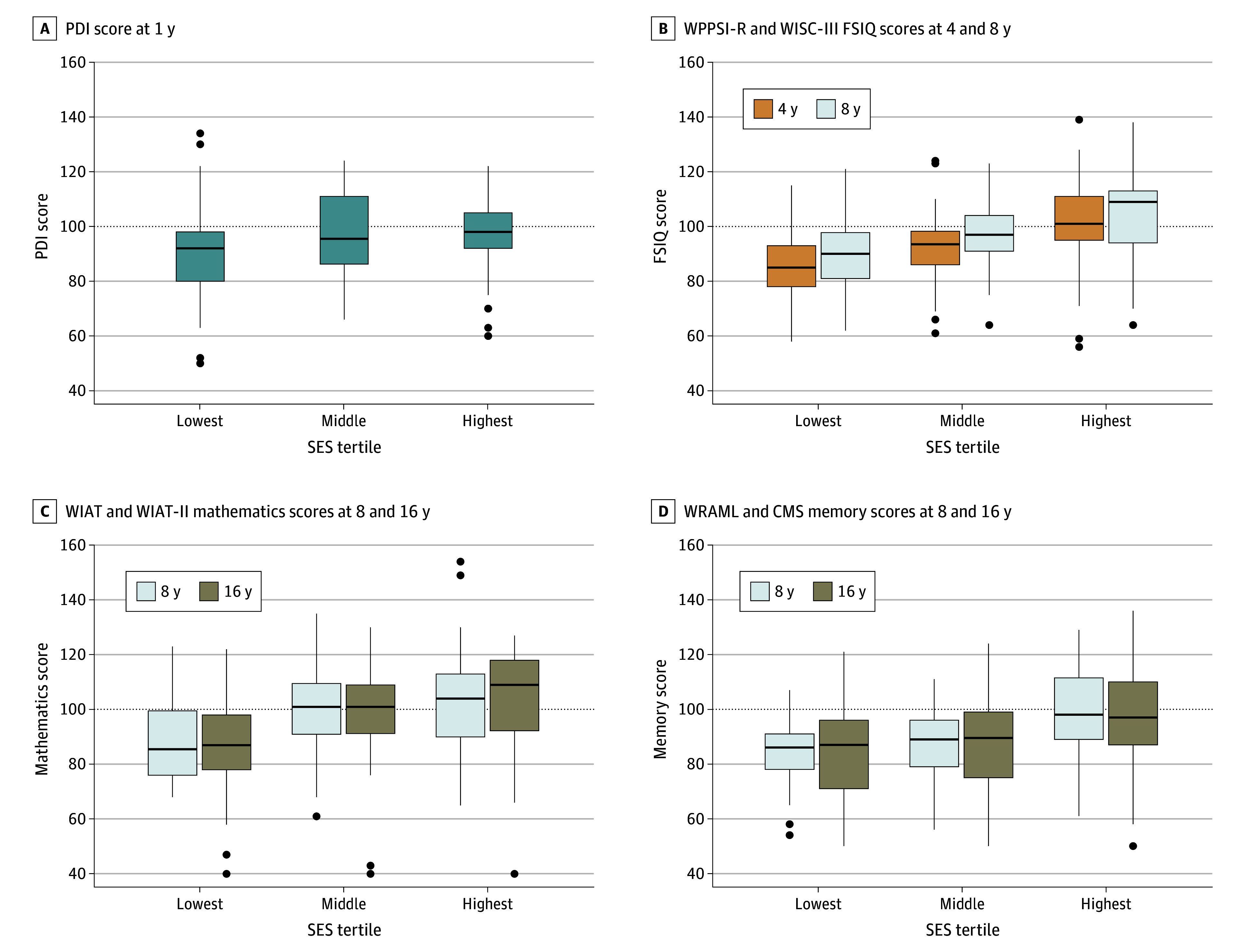
Selected Neurodevelopmental Outcomes by Socioeconomic Status (SES) Tertile The total number of observations was 144 for Psychomotor Development Index (PDI) (A), 155 for Wechsler Preschool and Primary Scale of Intelligence-Revised (WPPSI-R) and 154 for Wechsler Intelligence Scale for Children-Third Edition (WISC-III) Full Scale Intelligence Quotient (FSIQ) (B), 154 for Wechsler Individual Achievement Test (WIAT) and 140 WIAT-Second Edition II (WIAT-II) Mathematics (C), and 153 for Wide Range Assessment of Memory and Learning (WRAML) and 139 for Children’s Memory Scale (CMS) General Memory (D). eTable 1 in Supplement 1 provides the score ranges for these and other measures. Trends across SES tertiles were similar in age groups in panels B, C, and D (*P* > .32 on 2 *df* for each tertile, adjusting for birth and medical characteristics). Upper and lower ends of the boxes represent the 25th and 75th percentiles, respectively; horizontal line inside boxes represents the median; whiskers represent the IQR below the 25th percentile and above the 75th percentile; and circles above or below boxes represent data points outside the range of the whiskers.

Figure 1 depicts selected neurodevelopmental outcomes according to SES tertile and age at measurement, which were chosen because they were repeated across more than 1 time point. Patients in the lowest vs highest SES tertile had significantly lower FSIQ at 4 and 8 years of age, WIAT Mathematics score at 8 and 16 years of age, and Memory scores at ages 8 and 16 years. Where the same test was administered at consecutive ages, the magnitude of difference in test scores among SES tertiles was similar.

**Figure 2.**
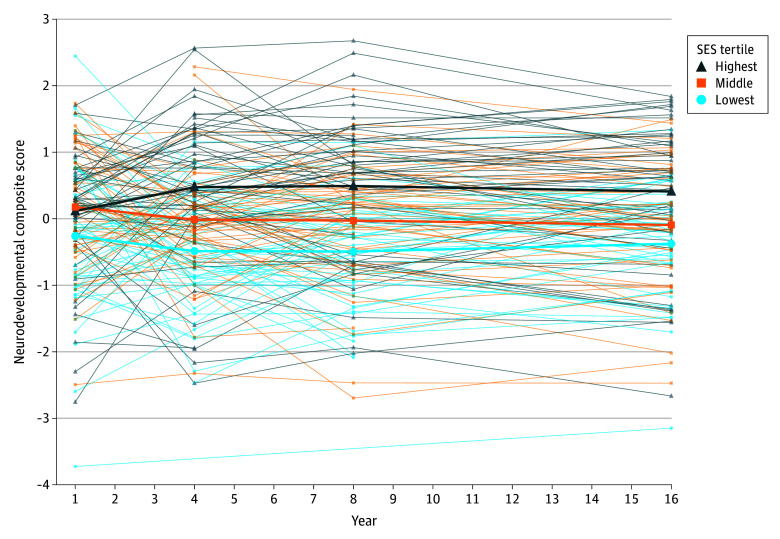
Neurodevelopmental Composite Scores by Socioeconomic Status (SES) Tertile and by Age Group Thick lines depict mean values of neurodevelopmental composite scores by SES tertile (53 observations in the lowest, 52 in the middle, and 55 in the highest) and age group. Thin lines connect values of neurodevelopmental composite scores for individuals. Interactions between age groups and SES tertiles are significant across ages 1, 4, 8, and 16 years (*P* = .02 on 6 *df*) but not across ages 4, 8, and 16 years (*P* = .50 on 4 *df*), adjusting for birth and medical characteristics due to the middle and highest SES tertiles being similar at age 1 year.

In neurodevelopmental composite findings from the PCA, the first principal component carried 72.5% of the variability in selected scores at age 1 year, 65.6% at age 4 years, 51.9% at age 8 years, and 62.4% at age 16 years. Participants in the highest and middle SES tertiles did not differ by mean (SD) neurodevelopmental composite scores at age 1 year, although scores in these combined SES tertiles were higher than scores in the lowest SES tertile (–0.26 [1.11] vs 0.14 [0.91]; *P* = .01) (Table 2, Figure 2). By age 4 years and continuing at ages 8 and 16 years, all 3 SES tertiles differed significantly in overall neurodevelopmental status, and there was no evidence of change in the magnitude of the association between SES and neurodevelopmental status over that time. Trends in neurodevelopmental composite outcomes as a function of continuous Hollingshead score were also found (eFigure 3 in Supplement 1).

### Latent Class Analysis

**Figure 3.**
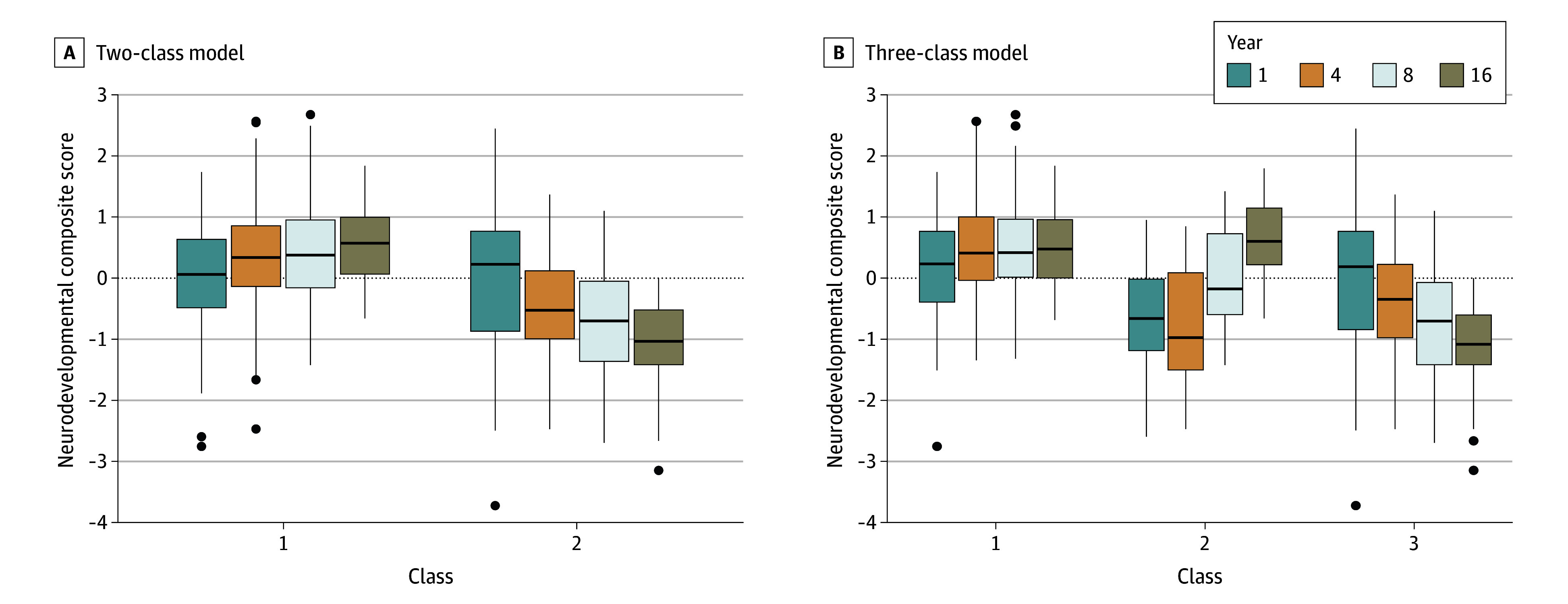
Neurodevelopmental Composite Scores by Latent Class Analysis The number of observations was 103 in class 1 (stable) and 57 in class 2 (declining) (A) and 85 in class 1 (stable), 20 in class 2 (improving), and 55 in class 3 (declining) (B). Upper and lower ends of the boxes represent the 25th and 75th percentiles, respectively; horizontal line inside boxes represent the median; whiskers represent the IQR below the 25th percentile and above the 75th percentile; and circles above or below boxes represent data points outside the range of the whiskers.

Longitudinal latent class analysis yielded 2- and 3-class solutions, both of which fit the data well (eTable 5 in Supplement 1 provides birth, medical, and sociodemographic characteristics by latent class). Given that this analysis included age-referenced scores and a standardized principal component, we anticipated relative stability of trajectories over time. Examining the 2-class model, whereas 1 class (stable; 103 [64%]) did maintain stability in neurodevelopmental status across the 4 time points, the second class (declining; 57 [36%]) seemed to decline progressively in neurodevelopmental status from age 1 year to 16 years. The 3-class model included the stable (85 [53%]) and declining (55 [34%]) groups and a smaller group of individuals (improving; 20 [13%]), most of whom were members of the stable group in the 2-class model, whose overall neurodevelopmental status improved over time (Figure 3). In the univariable analysis, latent class membership in both the 2- and 3-class models (stable vs improving vs declining class) was associated with younger maternal age at childbirth (29.6 [4.9] vs 29.1 [5.1] vs 26.6 [5.1] years; *P* = .002), lower maternal IQ (100.1 [11.1] vs 96.2 [11.0] vs 91.0 [14.1]; *P* < .001), and lower family SES (measured continuously) (40.9 [9.9] vs 35.8 [10.1] vs 35.2 [10.8]; *P* = .003). Latent class membership in the 3-class model was also associated with clinical seizures (eTable 5 in Supplement 1).

## Discussion

In this cohort study of the association of family SES with neurodevelopmental outcomes in patients with d-TGA, we found that lower SES was associated with worse outcomes across follow-up time points from ages 4 through 16 years after adjustment for preoperative patient, intraoperative management, and postoperative medical variables. SES-related disparities were evident on measures of general cognitive ability, language, executive function, memory, fine motor dexterity, and academic achievement. The magnitude of differences in overall neurodevelopmental status by SES tertile did not worsen over time for patients with d-TGA. However, compared with individuals whose neurodevelopmental status remained stable or improved, those who declined in neurodevelopmental status over time were more sociodemographically disadvantaged as measured by lower family SES, younger maternal age, and/or lower maternal IQ. Taken together, these findings support and extend those from prior studies and further attest to the pervasive and multifaceted role of SES in the development in individuals with CHD throughout the first 2 decades of life.

SES-related disparities in overall neurodevelopmental status were evident at around 1 year of age, specifically among infants with d-TGA from the lowest SES tertile compared with those from the middle and highest SES tertiles. We did not explore disparities at younger ages, and 1 year was younger than we had anticipated for SES-related differences to emerge. In the general population, neurodevelopmental trajectories seem to diverge along SES lines toward the end of the second year of life.^41,42^ Differences in receptive language by SES have been noted as early as age 15 months, and differences in baseline electroencephalographic activity have been recorded among infants from lower socioeconomic backgrounds as early as 6 to 9 months of age.^43^ However, data from typically developing children suggest that early-life electroencephalographic trajectories do not mediate the associations between family income and 24-month neurodevelopmental outcomes.^44^ Therefore, it is notable that the results of the present study reflect such early neurodevelopmental sensitivity among children with CHD who are from socioeconomically disadvantaged backgrounds and may imply a double threat, wherein brain dysmaturation and injury in CHD coupled with the stress of lower SES may be risk factors for an even earlier adverse outcome.

Findings from the longitudinal latent class analysis provide further evidence of the increasingly deleterious implications of social disadvantage for neurodevelopmental trajectories from toddlerhood to adolescence. Whereas stable to increasing average growth in neurodevelopment from 1 year to 16 years of age was more common among individuals in the middle and highest SES tertiles, those in the lowest SES tertile (and those whose mothers were younger at the time of childbirth and/or had lower IQ status) were more likely to show a decline in their relative position within the distribution. We hypothesized that this widening gap may be associated with slower neurodevelopmental growth among patients in the lowest SES tertile compared with patients in the middle or highest tertiles. These data highlighted that needs for individualized services are likely to be greater and increasingly so over time in patients with lower SES, bolstering society recommendations for periodic neurodevelopmental surveillance, monitoring, and evaluation throughout childhood and adolescence.^1,3^

As a proxy for a host of neurodevelopmentally meaningful factors, including food insecurity, poor nutrition, harmful environmental exposures, toxic stress, violence, trauma, reduced access to health care and prenatal care, and housing instability, lower SES has the potential to undermine brain development in many complex and persistent ways.^5,45,46,47,48^ In the general population, lower SES has been linked with multiple indicators of atypical brain structure and function, including reduced cortical and subcortical volumes, reduced cortical thickness and surface area, and differences in neural activation patterns during the performance of a variety of cognitive tasks.^5,48^

The effects of social disadvantage are likely to be amplified in children and families with critical CHD. Children from lower socioeconomic backgrounds are less likely to receive a prenatal CHD diagnosis,^7,11^ which in turn is associated with an increased likelihood of sustaining preoperative brain injury^49^ and worse neurodevelopmental outcomes.^50^ Moreover, children with CHD who lack private insurance are less likely to attend cardiac neurodevelopmental follow-up after surgery,^51^ which may decrease their access to recommended neurodevelopmental care per the American Heart Association Scientific Statement and Cardiac Neurodevelopmental Outcome Collaborative recommendations.^1,3,52,53^

The findings of this study underscored the need for early interventions aimed at mitigating the adverse implications of low SES for neurodevelopmental outcomes in children with critical CHD. Given that disparities in neurodevelopmental outcomes were evident quite early, policy interventions should prioritize early childhood programs, such as universal preschool, which can provide developmental support during formative years. Access to neurodevelopmental screening and therapy may be facilitated in families with lower SES by creating more accessible pathways for follow-up care, including telehealth options, extended clinic hours, or transportation assistance to alleviate the burden of travel and work-related conflicts. For families with limited means to travel or with competing priorities, targeted outreach initiatives, such as home visits, can help bridge gaps in neurodevelopmental care. Additionally, given the association of younger maternal age and lower maternal IQ with neurodevelopmental status declines, enhanced parental education in these groups may improve engagement.

### Strengths and Limitations

This study has several strengths. They include the prospective longitudinal design, excellent participant retention, and reliance on in-person administration of well-validated, performance-based assessment measures.

The findings should be interpreted within the context of study limitations. Participants were predominantly White individuals and underwent the ASO at a single center. The results cannot be generalized to individuals with genetic syndromes or extracardiac anomalies, as these groups were excluded from the study. Participants were followed up for neurodevelopmental outcomes from birth; thus, families may have had heightened sensitivity to the need for support services; however, if true, this would have reduced SES-related differences if families with lower SES accessed more services than they otherwise would have because of their BCAS participation. The BCAS sample, many of whom were referred for surgery to the center from other parts of the US, is likely an underrepresentation of very low SES tertiles; however, such a bias would have reduced rather than enhanced the finding of SES-related differences. Nonetheless, future research must include samples that better represent the sociodemographic characteristics of the broader CHD population. Toward this goal, it will be necessary to implement deliberate recruitment strategies to make study participation more feasible and less of a burden for families (eg, convenient scheduling of evaluations, childcare for siblings, and adequate reimbursement for participation and travel), along with concerted efforts to improve organizational health literacy within hospitals and research centers.^54^

This study did not include a longitudinal control group, but population norms were available for developmental testing. We were unable to include neighborhood-level SES variables in the analyses; evidence suggests that neighborhood-level SES factors account for unique variance in brain and behavioral outcomes in the general population,^55^ suggesting that a more comprehensive conceptualization of SES will be important for future research. Moreover, whereas the data demonstrate an apparent mitigation of neurodevelopmental risk associated with higher (vs lower) family SES among individuals with d-TGA, healthy individuals without CHD but with similarly high SES would be expected to score even higher on standardized measures, perhaps upward of a full SD above the population mean by adolescence.^41^ We cannot exclude the possibility that children with d-TGA from the highest socioeconomic backgrounds exhibit neurodevelopmental weaknesses compared with children without CHD and with high SES.

## Conclusions

The combination of d-TGA and lower SES was associated with significantly worse neurodevelopmental outcomes beginning as early as 1 year of age and continuing throughout adolescence. Individuals at a greater disadvantage because of lower family SES, younger maternal age at childbirth, and/or lower maternal IQ were at an increased risk for declines in neurodevelopmental status over time. Efforts to support children and families with CHD and lower SES must begin early, ideally at the time of diagnosis. Implementation of effective strategies for improving access to neurodevelopmental monitoring and intervention services for children with CHD from lower socioeconomic backgrounds is also necessary.

## Notes

Supplement 1. **eTable 1.** Neurodevelopmental Measures Administered and Associated Neurobehavioral Domains Assessed in the Boston Circulatory Arrest Study **eTable 2.** Comparison of Neurodevelopmental Outcomes at Each Year by SES Tertile **eTable 3.** Comparison of Neurodevelopmental Outcomes at Each Year by Maternal IQ Tertile **eTable 4.** Comparison of Neurodevelopmental Outcomes at Each Year by Maternal Education Category **eTable 5.** Birth, Medical, and Sociodemographic Characteristics by Latent Class **eFigure 1.** Flow Diagram Depicting Patient Follow-Up at Years 1, 4, 8, and 16 **eFigure 2.** Plots of Neurodevelopmental Composite Scores by Maternal IQ Tertile (A) or Maternal Education (B) and by Subject Across Age Groups **eFigure 3.** Relationship Between Neurodevelopmental Composite and Continuous Hollingshead Score at Each Year

Supplement 2.
Data Sharing Statement
