# Supplementary material for: Family Socioeconomic Status and Neurodevelopment Among Patients With Dextro-Transposition of the Great Arteries
Source: JAMA Netw Open. 2024 Nov 19;7(11):e2445863. doi: 10.1001/jamanetworkopen.2024.45863 (PMC11577140; doi:10.1001/jamanetworkopen.2024.45863)
Supplement: Supplement 1. — eTable 1. Neurodevelopmental Measures Administered and Associated Neurobehavioral Domains Assessed in the Boston Circulatory Arrest Study eTable 2. Comparison of Neurodevelopmental Outcomes at Each Year by SES Tertile eTable 3. Comparison of Neurodevelopmental Outcomes at Each Year by Maternal IQ Tertile eTable 4. Comparison of Neurodevelopmental Outcomes at Each Year by Maternal Education Category eTable 5. Birth, Medical, and Sociodemographic Characteristics by Latent Class eFigure 1. Flow Diagram Depicting Patient Follow-Up at Years 1, 4, 8, and 16 eFigure 2. Plots of Neurodevelopmental Composite Scores by Maternal IQ Tertile (A) or Maternal Education (B) and by Subject Across Age Groups eFigure 3. Relationship Between Neurodevelopmental Composite and Continuous Hollingshead Score at Each Year [file jamanetwopen-e2445863-s001.pdf]

Supplemental Online Content

Cassidy AR, Rofeberg V, Bucholz EM, Bellinger DC, Wypij D, Newburger JW. Family socioeconomic status and neurodevelopment among patients with dextro-transposition of the great arteries. *JAMA Netw Open*. 2024;7(11):e2445863. doi:10.1001/jamanetworkopen.2024.45863

eTable 1. Neurodevelopmental Measures Administered and Associated Neurobehavioral Domains Assessed in the Boston Circulatory Arrest Study

eTable 2. Comparison of Neurodevelopmental Outcomes at Each Year by SES Tertile

eTable 3. Comparison of Neurodevelopmental Outcomes at Each Year by Maternal IQ Tertile

eTable 4. Comparison of Neurodevelopmental Outcomes at Each Year by Maternal Education Category

eTable 5. Birth, Medical, and Sociodemographic Characteristics by Latent Class

eFigure 1. Flow Diagram Depicting Patient Follow-Up at Years 1, 4, 8, and 16

eFigure 2. Plots of Neurodevelopmental Composite Scores by Maternal IQ Tertile (A) or Maternal Education (B) and by Subject Across Age Groups

eFigure 3. Relationship Between Neurodevelopmental Composite and Continuous Hollingshead Score at Each Year

This supplemental material has been provided by the authors to give readers additional information about their work.

© 2024 Cassidy AR et al. *JAMA Network Open*

eTable 1. Neurodevelopmental Measures Administered and Associated Neurobehavioral Domains Assessed in the Boston Circulatory Arrest Study

Study Time Point	Measure	Score Type	Score Range	Neurobehavioral Domains Assessed	Are Higher Scores Indicative of Better or Worse Functioning?
1 Year	Bayley Scales of Infant and Toddler Development	Standard	50-150	General cognitive and motor abilities	Better
4 Years	Wechsler Preschool & Primary Scale of Intelligence-Revised (WPPSI-R)	Standard	41-160	General cognitive ability<br>Verbal IQ<br>Performance IQ	Better
	Receptive One-Word Picture Vocabulary Test	Standard	55-145	Receptive vocabulary	Better
	Expressive One-Word Picture Vocabulary Test	Standard	48-145	Expressive vocabulary	Better
	Grooved Pegboard	Time (seconds)	0-120	Fine motor dexterity	Worse
8 Years	Wechsler Intelligence Scale for Children-Third Edition (WISC-III)	Standard	40-160	General cognitive ability<br>Verbal IQ<br>Performance IQ	Better
	Errors of Commission (Test of Variables of Attention, TOVA)	Percentage	0-100	Sustained attention and inhibitory control	Worse
	Digit Span (WISC-III)	Scaled	1-19	Auditory attention and working memory	Better
	Trail-Making Test (Trails B minus Trails A)	Time (seconds)	-120-360	Cognitive flexibility	
	Wide Range Assessment of Memory and Learning (WRAML)	Standard	47-155	Memory (composite)	Better
	Grooved Pegboard	Time (seconds)	0-360	Fine motor dexterity	Worse
	Wechsler Individual Achievement Test (WIAT)	Standard	40-160	Reading<br>Mathematics	Better
16 Years	Delis-Kaplan Executive Function System (D-KEFS): Verbal Fluency, Design Fluency, Word Context, Sorting, Tower, Color-Word Interference Test (Inhibition trial), Trail Making Test (Letter-Number Switching trial)	Scaled	1-19	Executive function (composite)<br>Inhibitory control<br>Cognitive flexibility	Better
	Children's Memory Scale (CMS)	Standard	50-150	Memory (composite)	Better
	Numbers (CMS)	Scaled	1-19	Auditory attention and working memory	Better
	Global Executive Composite (Behavior Rating Inventory of Executive Function, BRIEF)	T	29-104	Everyday executive function skills (parent rating)	Worse
	Wechsler Individual Achievement Test-Second Edition (WIAT-II)	Standard	40-160	Reading<br>Mathematics	Better

Mean (SD): Standard score = 100 (15); Scaled score = 10 (3); T-score = 50 (10)

© 2024 Cassidy AR et al. *JAMA Network Open*

eTable 2. Comparison of Neurodevelopmental Outcomes at Each Year by SES Tertile

Outcome	Middle vs. Lowest	Highest vs. Lowest	Middle vs. Highest
1 Year			
•Psychomotor Development Index	0.5 [0.0, 0.9]	0.3 [-0.1, 0.7]	-0.2 [-0.6, 0.2]
•Mental Development Index	0.4 [0.0, 0.8]	0.4 [0.0, 0.8]	0.0 [-0.4, 0.4]
NDC-1	0.5 [0.1, 0.9]	0.4 [0.0, 0.8]	-0.1 [-0.5, 0.3]
4 Years			
•Full Scale IQ	0.5 [0.1, 0.8]	1.1 [0.7, 1.4]	0.6 [0.2, 0.9]
Verbal IQ	0.3 [0.0, 0.7]	1.0 [0.6, 1.3]	0.6 [0.3, 1.0]
Performance IQ	0.6 [0.2, 0.9]	0.9 [0.6, 1.3]	0.4 [0.0, 0.7]
Receptive One-Word Picture Vocabulary	0.6 [0.2, 1.0]	1.0 [0.5, 1.4]	0.3 [-0.1, 0.7]
•Expressive One-Word Picture Vocabulary	0.5 [0.1, 0.9]	1.0 [0.6, 1.4]	0.5 [0.1, 0.9]
•Grooved Pegboard	-0.3 [-0.7, 0.1]	-0.3 [-0.7, 0.1]	0.0 [-0.4, 0.3]
NDC-4	0.5 [0.1, 0.9]	1.0 [0.6, 1.3]	0.5 [0.1, 0.8]
8 Years			
•Full Scale IQ	0.6 [0.2, 0.9]	1.0 [0.7, 1.4]	0.5 [0.1, 0.8]
Verbal IQ	0.5 [0.1, 0.9]	1.0 [0.6, 1.4]	0.5 [0.1, 0.8]
Performance IQ	0.5 [0.1, 0.9]	0.9 [0.5, 1.2]	0.4 [0.1, 0.7]
•TOVA Errors of Commission	-0.2 [-0.7, 0.2]	-0.5 [-0.9, -0.1]	-0.3 [-0.7, 0.1]
•Digit Span	0.3 [-0.1, 0.6]	0.5 [0.1, 0.8]	0.2 [-0.1, 0.6]
•Trail-Making B-A	-0.1 [-0.5, 0.3]	-0.4 [-0.8, 0.0]	-0.3 [-0.7, 0.1]
•WRAML Memory Screening Index	0.2 [-0.2, 0.6]	0.9 [0.6, 1.3]	0.8 [0.4, 1.1]
•Grooved Pegboard	-0.4 [-0.8, 0.0]	-0.6 [-0.9, -0.2]	-0.2 [-0.6, 0.2]
•WIAT Reading	0.6 [0.2, 1.0]	0.9 [0.5, 1.3]	0.4 [0.0, 0.7]
•WIAT Mathematics	0.8 [0.4, 1.3]	1.0 [0.6, 1.5]	0.2 [-0.2, 0.6]
NDC-8	0.6 [0.2, 0.9]	1.0 [0.7, 1.4]	0.5 [0.1, 0.8]
16 Years			
DKEFS Average	0.4 [0.1, 0.6]	0.5 [0.3, 0.8]	0.2 [-0.1, 0.5]
•DKEFS Inhibition	0.2 [-0.3, 0.7]	0.6 [0.1, 1.1]	0.5 [0.0, 0.9]
•DKEFS Number-Letter Switching	0.0 [-0.4, 0.5]	0.5 [0.1, 1.0]	0.5 [0.0, 0.9]
•CMS General Memory	0.4 [-0.2, 0.9]	0.9 [0.3, 1.4]	0.5 [0.0, 1.0]
•CMS Numbers Total Score	0.2 [-0.3, 0.7]	0.4 [0.0, 0.9]	0.2 [-0.2, 0.7]
BRIEF-Parent Global	0.2 [-0.4, 0.7]	-0.5 [-1.0, 0.0]	-0.6 [-1.1, -0.1]
•WIAT-II Reading	0.6 [0.2, 1.0]	1.1 [0.7, 1.5]	0.5 [0.1, 0.9]
•WIAT-II Mathematics	0.9 [0.3, 1.4]	1.1 [0.6, 1.6]	0.2 [-0.3, 0.8]
NDC-16	0.4 [0.0, 0.8]	0.8 [0.4, 1.2]	0.4 [0.0, 0.8]

Values are reported as standardized mean difference comparing groups, the effect size relative to 1 SD based on test parameters when available or else based on SD estimated from our data, with [95% confidence interval]. Standardized mean differences and confidence intervals were calculated by analysis of covariance adjusting for selected birth and medical characteristics. SES = socioeconomic status, NDC = neurodevelopmental composite, IQ = intelligence quotient, TOVA = Test of Variables of Attention, WRAML = Wide Range Assessment of Memory and Learning, WIAT = Wechsler Individual Achievement Test, DKEFS = Delis-Kaplan Executive Function System, CMS = Children's Memory Scale, BRIEF = Behavior Rating Inventory of Executive Function.
•Indicates contribution to the NDC.

© 2024 Cassidy AR et al. *JAMA Network Open*

eTable 3. Comparison of Neurodevelopmental Outcomes at Each Year by Maternal IQ Tertile

Outcome	Lowest (n = 50)	Middle (n = 47)	Highest (n = 53)	Adjusted P-value	Middle vs. Lowest	Highest vs. Lowest	Middle vs. Highest
1 Year							
•Psychomotor Development Index	92.8 (16.5)	96.7 (15.9)	94.9 (15.2)	0.57	0.2 [-0.2, 0.7]	0.1 [-0.3, 0.5]	-0.1 [-0.6, 0.3]
•Mental Development Index	99.8 (17.3	106.8 (14.1)	106.7 (14.4)	0.07	0.5 [0.0, 0.9]	0.4 [0.0, 0.9]	0.0 [-0.5, 0.4]
NDC-1	-0.26 (1.06)	0.15 (0.98)	0.08 (0.93)	0.14	0.4 [0.0, 0.8]	0.3 [-0.1, 0.7]	-0.1 [-0.5, 0.3]
4 Years							
•Full Scale IQ	86.7 (13.6)	94.8 (14.6)[a]	96.4 (14.3)[a]	0.01	0.5 [0.1, 0.9]	0.6 [0.2, 1.0]	0.1 [-0.3, 0.5]
Verbal IQ	90.2 (12.9)	97.5 (14.1)	98.2 (16.5)[a]	0.02	0.5 [0.1, 0.9]	0.5 [0.1, 0.9]	0.0 [-0.3, 0.4]
Performance IQ	85.7 (13.9)	93.0 (15.3)	95.6 (12.4)[a]	0.01	0.4 [0.0, 0.8]	0.6 [0.2, 1.0]	0.2 [-0.2, 0.5]
Receptive One-Word Picture Vocabulary	88.0 (15.6)	100.7 (13.9)[a]	102.3 (13.8)[a]	<0.001	0.8 [0.4, 1.3]	0.9 [0.5, 1.3]	0.1 [-0.3, 0.5]
•Expressive One-Word Picture Vocabulary	85.4 (11.9)	95.1 (14.9)[a]	97.9 (16.9)[a]	<0.001	0.8 [0.4, 1.2]	0.9 [0.5, 1.3]	0.1 [-0.3, 0.5]
•Grooved Pegboard	94.5 (39.6)	93.1 (39.1)	86.1 (38.5)	0.53	0.1 [-0.3, 0.5]	-0.1 [-0.5, 0.3]	-0.2 [-0.6, 0.2]
NDC-4	-0.39 (0.84)	0.14 (0.96)[a]	0.32 (1.04)[a]	0.002	0.5 [0.1, 0.9]	0.7 [0.3, 1.1]	0.2 [-0.2, 0.5]
8 Years							
•Full Scale IQ	90.9 (14.0)	99.2 (13.7)[a]	102.8 (15.8)[a]	<0.001	0.5 [0.1, 0.9]	0.8 [0.4, 1.2]	0.2 [-0.1, 0.6]
Verbal IQ	93.1 (13.5)	102.8 (15.2)[a]	105.2 (17.8)[a]	<0.001	0.7 [0.2, 1.1]	0.8 [0.4, 1.2]	0.2 [-0.2, 0.6]
Performance IQ	90.3 (15.3)	95.6 (13.2)	99.8 (13.4)[a]	0.01	0.3 [-0.1, 0.7]	0.6 [0.2, 1.0]	0.3 [-0.1, 0.7]
•TOVA Errors of Commission	13.1 (17.8)	10.6 (13.2)	10.8 (14.2)	0.46	-0.3 [-0.7, 0.2]	-0.2 [-0.6, 0.2]	0.1 [-0.3, 0.5]
•Digit Span	8.4 (2.7)	8.5 (2.4)	9.6 (2.7)[a]	0.03	0.1 [-0.3, 0.5]	0.4 [0.1, 0.8]	0.4 [0.0, 0.7]
•Trail-Making B-A	69.9 (68.3)	53.0 (31.7)	48.8 (53.0)	0.17	-0.3 [-0.7, 0.2]	-0.4 [-0.8, 0.0]	-0.1 [-0.5, 0.3]
•WRAML Memory Screening Index	83.2 (14.5)	91.2 (13.0)[a]	96.1 (15.1)[a]	<0.001	0.6 [0.2, 1.0]	0.9 [0.5, 1.3]	0.3 [-0.1, 0.7]
•Grooved Pegboard	105.1 (38.5)	96.3 (19.2)	94.2 (24.7)	0.10	-0.3 [-0.7, 0.1]	-0.4 [-0.8, 0.0]	-0.1 [-0.5, 0.3]
•WIAT Reading	89.0 (13.9)	95.9 (14.3)	101.6 (15.1)[a]	<0.001	0.5 [0.1, 0.9]	0.9 [0.5, 1.3]	0.4 [0.0, 0.8]
•WIAT Mathematics	90.4 (18.4)	97.0 (14.5)	102.0 (17.4)[a]	0.005	0.5 [0.0, 1.0]	0.8 [0.3, 1.3]	0.3 [-0.1, 0.8]
NDC-8	-0.43 (1.00)	0.07 (0.79)[a]	0.40 (1.03)[a]	<0.001	0.5 [0.1, 0.9]	0.9 [0.5, 1.3]	0.3 [0.0, 0.7]
16 Years							
DKEFS Average	8.4 (2.3)	9.5 (1.6)[a]	9.3 (2.3)	0.02	0.4 [0.1, 0.7]	0.3 [0.0, 0.6]	-0.1 [-0.4, 0.2]
•DKEFS Inhibition	7.9 (3.3)	7.2 (3.6)	9.2 (3.5)[b]	0.03	-0.2 [-0.7, 0.3]	0.5 [0.0, 1.0]	0.6 [0.2, 1.1]
•DKEFS Number-Letter Switching	7.7 (3.5)	7.9 (3.4)	9.1 (3.2)	0.04	0.1 [-0.4, 0.6]	0.6 [0.1, 1.0]	0.5 [0.0, 0.9]
•CMS General Memory	83.3 (19.8)	94.6 (15.9)[a]	92.3 (19.3)	0.01	0.8 [0.3, 1.4]	0.6 [0.1, 1.2]	-0.2 [-0.7, 0.3]
•CMS Numbers Total Score	7.4 (3.6)	7.6 (3.5)	8.6 (3.0)	0.16	0.1 [-0.5, 0.6]	0.4 [-0.1, 0.9]	0.4 [-0.1, 0.8]
BRIEF-Parent Global	55.3 (13.8)	53.5 (9.6)	56.6 (12.6)	0.45	-0.2 [-0.8, 0.3]	0.1 [-0.4, 0.7]	0.3 [-0.2, 0.9]

© 2024 Cassidy AR et al. *JAMA Network Open*

•WIAT-II Reading	87.5 (16.3)	96.9 (13.4)[a]	100.8 (15.9)[a]	<0.001	0.7 [0.2, 1.1]	1.0 [0.6, 1.4]	0.3 [-0.1, 0.7]
•WIAT-II Mathematics	89.1 (21.7)	98.4 (16.2)	101.8 (19.6)[a]	0.01	0.6 [0.1, 1.2]	0.9 [0.3, 1.4]	0.2 [-0.3, 0.8]
NDC-16	-0.36 (1.04)	-0.01 (0.91)	0.31 (1.00)[a]	0.004	0.4 [0.0, 0.8]	0.7 [0.3, 1.1]	0.3 [-0.1, 0.7]

Values are reported as mean (standard deviation) or standardized mean difference comparing groups with [95% confidence interval]. P-values with 2 degrees of freedom comparing maternal IQ tertiles, standardized mean differences, and confidence intervals were calculated by analysis of covariance adjusting for selected birth and medical characteristics. Lowest maternal IQ tertile ranges from 56 to 92, middle maternal IQ tertile ranges from 93 to 103, and highest maternal IQ tertile ranges from 104 to 123. IQ = intelligence quotient, NDC = neurodevelopmental composite, TOVA = Test of Variables of Attention, WRAML = Wide Range Assessment of Memory and Learning, WIAT = Wechsler Individual Achievement Test, DKEFS = Delis-Kaplan Executive Function System, CMS = Children's Memory Scale, BRIEF = Behavior Rating Inventory of Executive Function.

[a]P < 0.0167 vs. lowest maternal IQ tertile adjusting for selected birth and medical characteristics.

[b]P < 0.0167 vs. middle maternal IQ tertile adjusting for selected birth and medical characteristics.

•Indicates contribution to the NDC.

© 2024 Cassidy AR et al. *JAMA Network Open*

eTable 4. Comparison of Neurodevelopmental Outcomes at Each Year by Maternal Education Category

Outcome	Lowest (n = 45)	Middle (n = 39)	Highest (n = 80)	Adjusted P-value	Middle vs. Lowest	Highest vs. Lowest	Middle vs. Highest
1 Year							
•Psychomotor Development Index	97.2 (15.3)	91.4 (17.3)	95.2 (15.3)	0.28	-0.4 [-0.9, 0.1]	-0.2 [-0.6, 0.2]	0.2 [-0.2, 0.6]
•Mental Development Index	102.1 (18.0)	102.2 (17.1)	107.5 (12.8)	0.19	0.1 [-0.4, 0.6]	0.4 [-0.1, 0.8]	0.3 [-0.2, 0.7]
NDC-1	0.01 (1.11)	-0.22 (1.11)	0.12 (0.87)	0.42	-0.2 [-0.6, 0.3]	0.1 [-0.3, 0.5]	0.3 [-0.1, 0.7]
4 Years							
•Full Scale IQ	87.4 (13.6)	87.6 (10.6)	97.9 (15.4)[ab]	<0.001	0.0 [-0.4, 0.4]	0.7 [0.3, 1.0]	0.7 [0.3, 1.1]
Verbal IQ	89.7 (12.3)	91.1 (11.8)	100.0 (16.3)[ab]	<0.001	0.1 [-0.4, 0.5]	0.7 [0.3, 1.0]	0.6 [0.2, 1.0]
Performance IQ	87.5 (15.2)	86.6 (11.6)	96.1 (13.9)[ab]	<0.001	-0.1 [-0.5, 0.3]	0.6 [0.2, 0.9]	0.7 [0.3, 1.0]
Receptive One-Word Picture Vocabulary	90.7 (15.7)	92.5 (17.2)	102.4 (12.9)[ab]	<0.001	-0.1 [-0.5, 0.4]	0.6 [0.2, 1.0]	0.7 [0.3, 1.1]
•Expressive One-Word Picture Vocabulary	87.8 (15.3)	88.8 (14.1)	96.5 (15.7)[ab]	0.01	0.0 [-0.5, 0.5]	0.5 [0.1, 0.9]	0.6 [0.2, 1.0]
•Grooved Pegboard	89.8 (39.0)	101.8 (42.3)	88.0 (37.9)	0.10	0.4 [-0.1, 0.8]	0.0 [-0.4, 0.3]	-0.4 [-0.8, 0.0]
NDC-4	-0.27 (1.0)	-0.33 (0.84)	0.30 (1.01)[ab]	<0.001	-0.1 [-0.6, 0.3]	0.5 [0.2, 0.9]	0.7 [0.3, 1.0]
8 Years							
•Full Scale IQ	91.3 (14.0)	93.6 (12.8)	101.9 (15.6)[ab]	<0.001	0.1 [-0.3, 0.5]	0.7 [0.3, 1.0]	0.6 [0.2, 1.0]
Verbal IQ	93.4 (14.6)	97.6 (14.3)	104.1 (17.4)[a]	0.002	0.2 [-0.3, 0.7]	0.7 [0.3, 1.1]	0.5 [0.1, 0.9]
Performance IQ	90.4 (14.5)	90.6 (13.3)	99.4 (13.4)	<0.001	0.0 [-0.5, 0.4]	0.6 [0.2, 0.9]	0.6 [0.2, 1.0]
•TOVA Errors of Commission	15.1 (19.9)	11.4 (15.1)	9.9 (13.2)	0.21	-0.3 [-0.8, 0.2]	-0.4 [-0.8, 0.0]	-0.1 [-0.5, 0.3]
•Digit Span	8.1 (2.3)	8.8 (2.3)	9.3 (2.9)[a]	0.04	0.3 [-0.1, 0.7]	0.4 [0.1, 0.8]	0.2 [-0.2, 0.5]
•Trail-Making B-A	61.9 (51.0)	60.0 (63.7)	52.6 (48.6)	0.45	-0.1 [-0.6, 0.3]	-0.2 [-0.6, 0.1]	-0.1 [-0.5, 0.3]
•WRAML Memory Screening Index	86.4 (13.8)	83.9 (12.9)	94.8 (15.8)[ab]	<0.001	-0.2 [-0.6, 0.3]	0.5 [0.2, 0.9]	0.7 [0.3, 1.1]
•Grooved Pegboard	98.2 (27.9)	110.1 (41.1)	95.4 (22.2)[b]	0.01	0.4 [-0.1, 0.8]	-0.2 [-0.6, 0.2]	-0.6 [-1.0, -0.2]
•WIAT Reading	91.1 (15.7)	92.4 (13.0)	98.8 (15.1)[a]	0.01	0.1 [-0.3, 0.6]	0.6 [0.2, 1.0]	0.4 [0.0, 0.8]
•WIAT Mathematics	91.1 (16.7)	92.6 (14.1)	101.4 (17.6)[ab]	<0.001	0.1 [-0.4, 0.6]	0.7 [0.3, 1.2]	0.6 [0.1, 1.0]
NDC-8	-0.33 (0.89)	-0.27 (0.85)	0.30 (1.04)[ab]	0.002	0.1 [-0.4, 0.5]	0.7 [0.3, 1.0]	0.6 [0.2, 1.0]
16 Years							
DKEFS Average	8.6 (1.9)	8.5 (2.2)	9.5 (2.0)[b]	0.01	0.0 [-0.4, 0.3]	0.3 [0.1, 0.6]	0.4 [0.1, 0.7]
•DKEFS Inhibition	8.6 (2.7)	7.3 (3.8)	8.2 (3.8)	0.63	-0.3 [-0.9, 0.3]	-0.1 [-0.6, 0.4]	0.2 [-0.3, 0.7]
•DKEFS Number-Letter Switching	8.3 (3.4)	7.2 (3.6)	8.8 (3.0)[b]	0.04	-0.4 [-0.9, 0.2]	0.2 [-0.2, 0.7]	0.6 [0.1, 1.1]
•CMS General Memory	89.5 (20.3)	81.9 (15.8)	95.0 (17.4)[b]	0.004	-0.5 [-1.1, 0.1]	0.3 [-0.1, 0.8]	0.9 [0.4, 1.4]
•CMS Numbers Total Score	7.4 (3.0)	7.5 (3.4)	8.3 (3.5)	0.27	0.1 [-0.5, 0.6]	0.3 [-0.1, 0.8]	0.3 [-0.2, 0.8]

© 2024 Cassidy AR et al. *JAMA Network Open*

BRIEF-Parent Global	55.0 (12.5)	58.9 (11.3)	52.9 (12.1)	0.11	0.3 [-0.3, 0.9]	-0.3 [-0.8, 0.2]	-0.5 [-1.1, 0.0]
•WIAT-II Reading	91.5 (13.3)	90.2 (15.6)	100.3 (16.3)[ab]	<0.001	0.0 [-0.5, 0.5]	0.7 [0.3, 1.1]	0.7 [0.3, 1.1]
•WIAT-II Mathematics	89.4 (19.9)	92.9 (18.9)	102.3 (18.6)[ab]	0.002	0.2 [-0.4, 0.8]	0.9 [0.4, 1.4]	0.7 [0.1, 1.2]
NDC-16	-0.16 (0.92)	-0.34 (0.91)	0.24 (1.03)[b]	0.01	-0.1 [-0.6, 0.3]	0.5 [0.0, 0.9]	0.6 [0.2, 1.0]

Values are reported as mean (standard deviation) or standardized mean difference comparing groups with [95% confidence interval]. P-values with 2 degrees of freedom comparing maternal education categories, standardized mean differences, and confidence intervals were calculated by analysis of covariance adjusting for selected birth and medical characteristics. Lowest education level refers to high school or less, middle education level refers to some college, and highest education level refers to college or graduate school. NDC = neurodevelopmental composite, IQ = intelligence quotient, TOVA = Test of Variables of Attention, WRAML = Wide Range Assessment of Memory and Learning, WIAT = Wechsler Individual Achievement Test, DKEFS = Delis-Kaplan Executive Function System, CMS = Children's Memory Scale, BRIEF = Behavior Rating Inventory of Executive Function.

[a]P < 0.0167 vs. lowest maternal education category adjusting for selected birth and medical characteristics.

[b]P < 0.0167 vs. middle maternal education category adjusting for selected birth and medical characteristics.

•Indicates contribution to the NDC.

© 2024 Cassidy AR et al. *JAMA Network Open*

eTable 5. Birth, Medical, and Sociodemographic Characteristics by Latent Class

Characteristic	Two Class Model Class 1 Stable (n = 103)	Two Class Model Class 2 Declining (n = 57)	P-value	Three Class Model Class 1 Stable (n = 85)	Three Class Model Class 2 Improving (n = 20)	Three Class Model Class 3 Declining (n = 55)	P-value
Birth characteristics							
Gestational age at birth (wk)	39.8 (1.2)	39.8 (1.3)	0.88	39.8 (1.2)	39.9 (1.0)	39.7 (1.2)	0.94
Birth weight (kg)	3.5 (0.4)	3.6 (0.4)	0.34	3.5 (0.4)	3.5 (0.4)	3.6 (0.4)	0.22
White non-Hispanic	95 (92%)	48 (84%)	0.12	77 (91%)	18 (90%)	48 (87%)	0.82
Medical characteristics							
Ventricular septal defect	24 (23%)	14 (25%)	0.86	19 (22%)	5 (25%)	14 (25%)	0.91
Total DHCA time (min)	36 (21)	37 (23)	0.71	36 (21)	31 (22)	38 (23)	0.46
Total support time (min)	143 (27)	143 (37)	0.91	141 (27)	145 (30)	145 (37)	0.81
Time from first surgery to discharge (d)	11 (7)	12 (9)	0.36	11 (8)	11 (4)	12 (9)	0.75
Clinical seizure	4 (4%)	7 (12%)	0.06	1 (1%)	3 (15%)	7 (13%)	0.005
Sociodemographic characteristics							
Maternal age at birth (yr)	29.7 (4.8)	26.4 (5.1)	<0.001	29.6 (4.9)	29.1 (5.1)	26.6 (5.1)	0.002
Maternal education			0.13				0.27
High school or less	26 (25%)	17 (30%)		47 (55%)	9 (45%)	22 (40%)	
Some college	21 (20%)	18 (32%)		23 (27%)	5 (25%)	15 (27%)	
College or graduate school	56 (54%)	22 (39%)		15 (18%)	6 (30%)	18 (33%)	
Maternal IQ	99.6 (11.1)	90.6 (13.7)	<0.001	100.1 (11.1)	96.2 (11.0)	91.0 (14.1)	<0.001
Hollingshead score	40.3 (9.8)	34.8 (11.1)	0.002	40.9 (9.9)	35.8 (10.1)	35.2 (10.8)	0.003
SES tertile			0.06				0.05
Lowest	29 (28%)	24 (42%)		21 (25%)	10 (50%)	22 (40%)	
Middle	32 (31%)	20 (35%)		27 (32%)	5 (25%)	20 (36%)	
Highest	42 (21%)	13 (23%)		37 (44%)	5 (25%)	13 (24%)	

Values are reported as mean (standard deviation) or n (%). P-values to predict latent classes were calculated by logistic regression for the two class model and multinomial logistic regression for the three class model. DHCA=deep hypothermic circulatory arrest, IQ=intelligence quotient, SES=socioeconomic status.

© 2024 Cassidy AR et al. *JAMA Network Open*

eFigure 1. Flow Diagram Depicting Patient Follow-Up at Years 1, 4, 8, and 16

Participants who were deemed untestable did not have neurodevelopmental data available. Exclusion of neurodevelopmental data for pilot testing at year 1 were for infants evaluated under conditions that were not standardized in terms of time, location, and the children's condition. The National Institutes of Health Data and Safety Monitoring Committee for the trial approved our decision not to include these children in analyses of developmental end points.

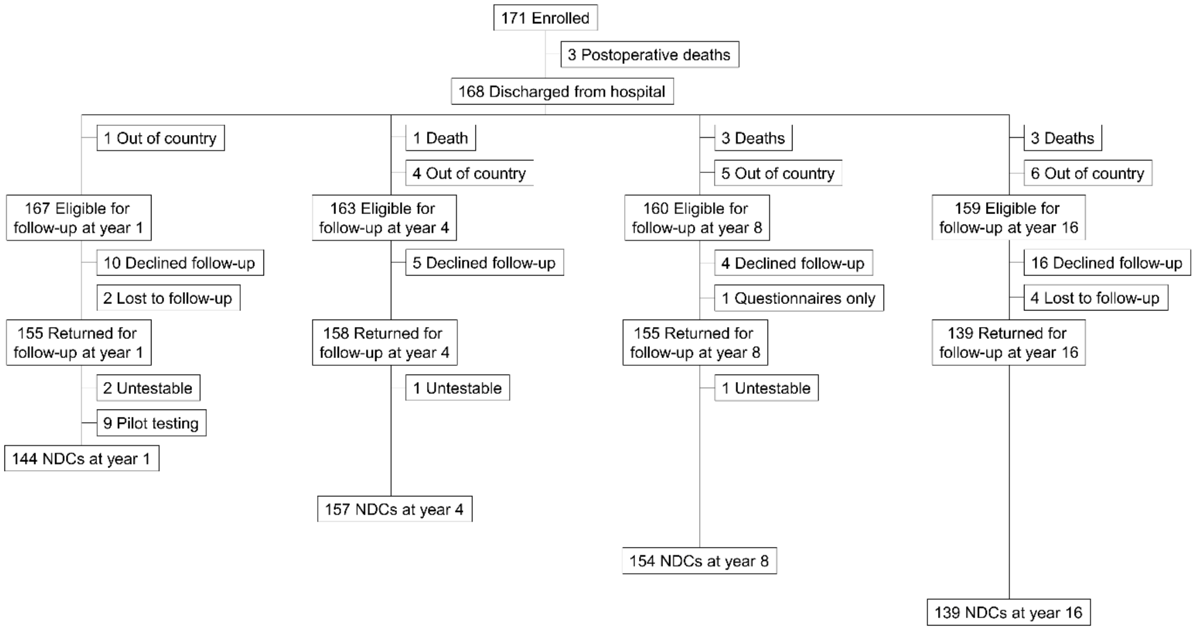

© 2024 Cassidy AR et al. *JAMA Network Open*

eFigure 2. Plots of Neurodevelopmental Composite Scores by Maternal IQ Tertile (A) or Maternal Education (B) and by Subject Across Age Groups

Thick lines depict mean values of neurodevelopmental composite scores by tertile by age group. Thin lines connect values of neurodevelopmental composite scores for individual subjects.

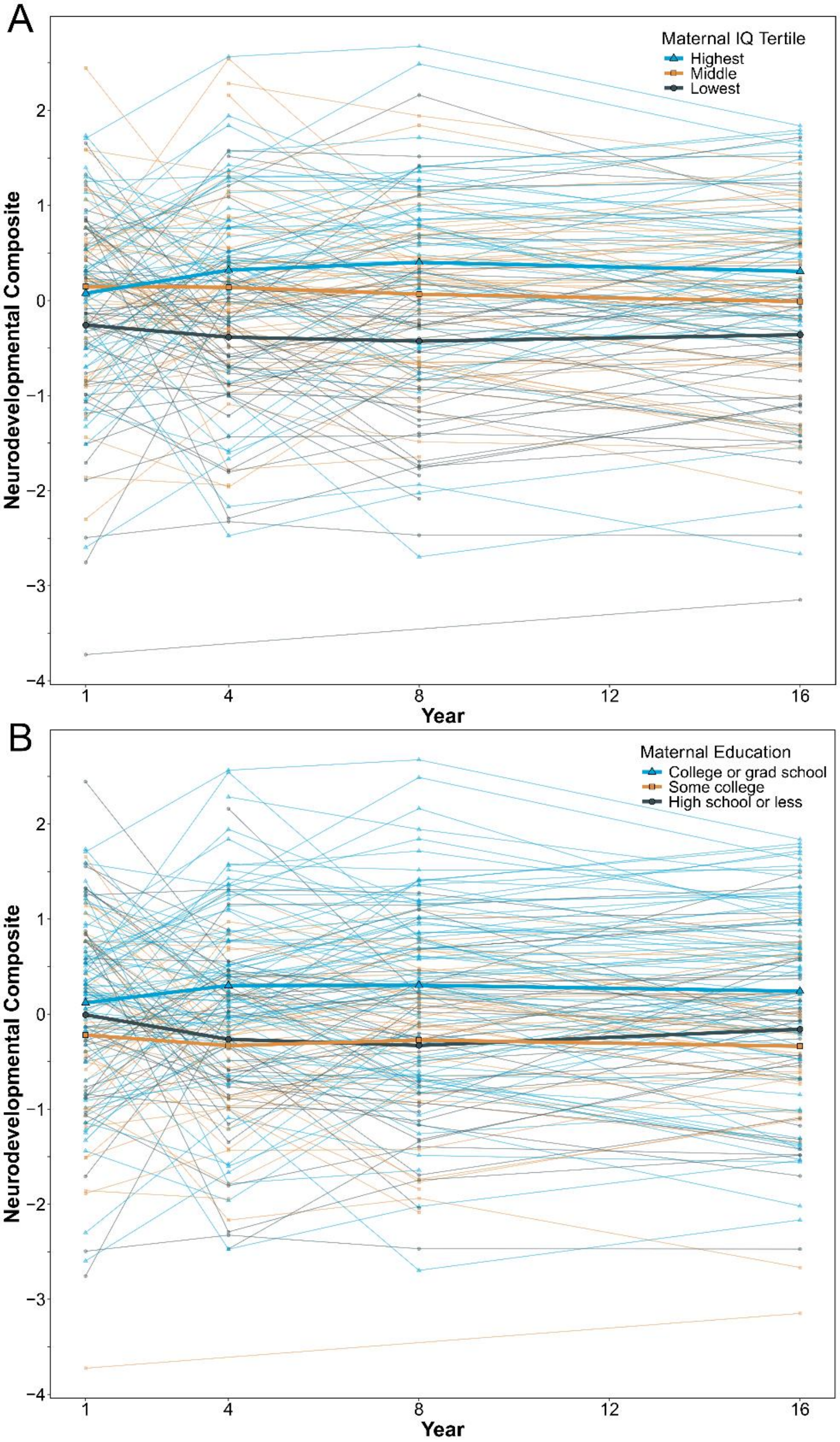

© 2024 Cassidy AR et al. *JAMA Network Open*

eFigure 3. Relationship Between Neurodevelopmental Composite and Continuous Hollingshead Score at Each Year

Blue lines depict linear fit with confidence bands, red lines depict the mean neurodevelopmental composite by SES tertile, and black lines depict natural cubic splines.

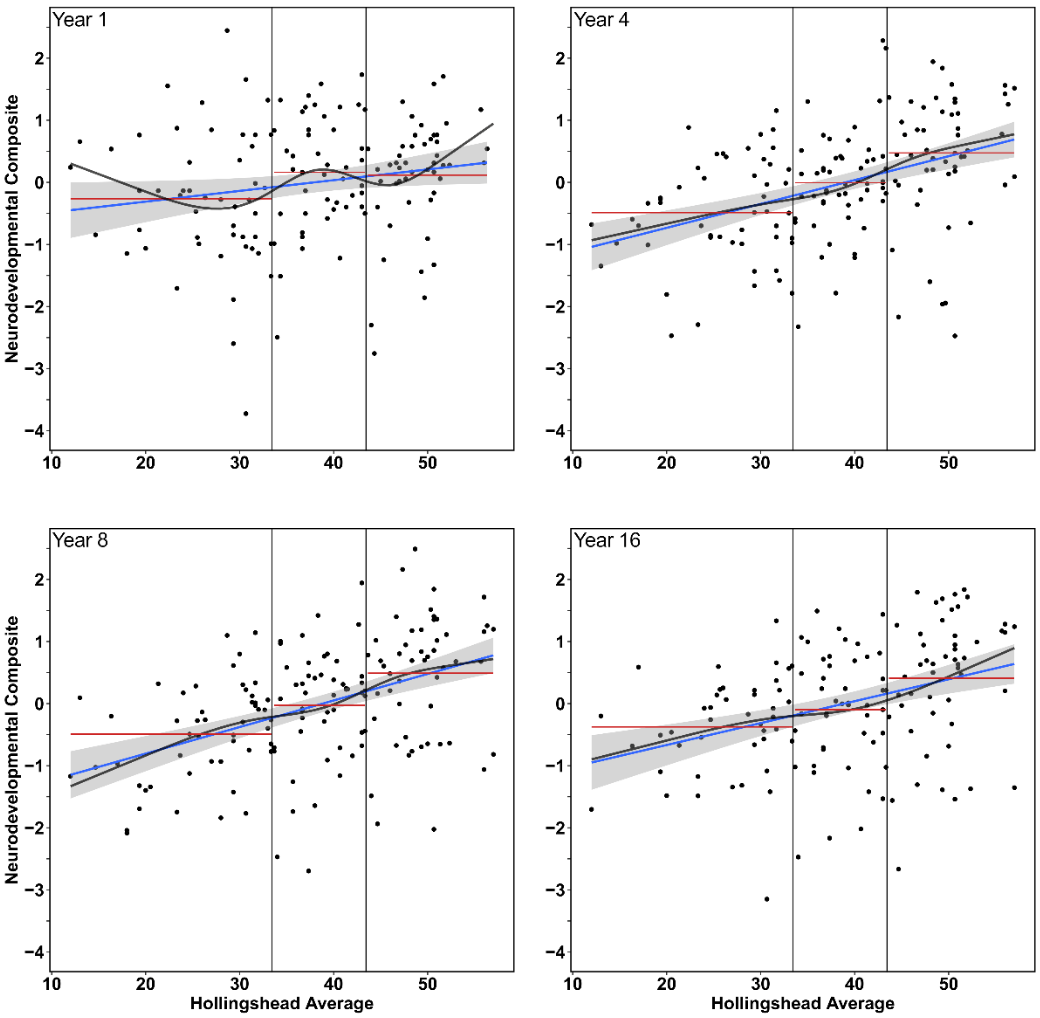

© 2024 Cassidy AR et al. *JAMA Network Open*
