# Supplementary material for: Family Socioeconomic Status and Neurodevelopment Among Patients With Dextro-Transposition of the Great Arteries
Source: JAMA Netw Open. 2024 Nov 19;7(11):e2445863. doi: 10.1001/jamanetworkopen.2024.45863 (PMC11577140; doi:10.1001/jamanetworkopen.2024.45863)
Supplement: Supplement 2. — Data Sharing Statement [file jamanetwopen-e2445863-s002.pdf]

Data Sharing Statement

Cassidy. Family Socioeconomic Status and Neurodevelopment Among Patients With Dextro-Transposition of the Great Arteries. *JAMA Netw Open*. Published November 19, 2024. doi:10.1001/jamanetworkopen.2024.45863

Data
Data available: Yes
Data types: Deidentified participant data, Other (please specify)
Additional Information: analytic methods, study materials
How to access data: Data requests should be sent to senior authors: Jane Newburger (jane.newburger@cardio.chboston.org) and David Wypij (david.wypij@cardio.chboston.org)
When available: With publication

Supporting Documents
Document types: None

Additional Information
Who can access the data: Researchers whose proposed use of the data has been approved
Types of analyses: For the purpose of reproducing the results or replicating the procedure
Mechanisms of data availability: After approval of a proposal, with a signed data access agreement
